# Multiplex Immunoassay of Lower Genital Tract Mucosal Fluid from Women Attending an Urban STD Clinic Shows Broadly Increased IL1ß and Lactoferrin

**DOI:** 10.1371/journal.pone.0019560

**Published:** 2011-05-10

**Authors:** Gregory T. Spear, Sabrina R. Kendrick, Hua Y. Chen, Tin T. Thomas, Mieoak Bahk, Robert Balderas, Santosh Ghosh, Aaron Weinberg, Alan L. Landay

**Affiliations:** 1 Department of Immunology/Microbiology, Rush University Medical Center, Chicago, Illinois, United States of America; 2 Department of Medicine, John Stroger Hospital of Cook County, Chicago, Illinois, United States of America; 3 Ruth M. Rothstein CORE Center, Chicago, Illinois, United States of America; 4 Division of Epidemiology and Biostatistics, University of Illinois, Chicago, Illinois, United States of America; 5 BD Biosciences, San Diego, California, United States of America; 6 Department of Biological Sciences, Case School of Dental Medicine, Cleveland, Ohio, United States of America; University of California Merced, United States of America

## Abstract

**Background:**

More than one million new cases of sexually transmitted diseases (STDs) occur each day. The immune responses and inflammation induced by STDs and other frequent non-STD microbial colonizations (i.e. Candida and bacterial vaginosis) can have serious pathologic consequences in women including adverse pregnancy outcomes, infertility and increased susceptibility to infection by other pathogens. Understanding the types of immune mediators that are elicited in the lower genital tract by these infections/colonizations can give important insights into the innate and adaptive immune pathways that are activated and lead to strategies for preventing pathologic effects.

**Methodology/Principal Findings:**

32 immune mediators were measured by multiplexed immunoassays to assess the immune environment of the lower genital tract mucosa in 84 women attending an urban STD clinic. IL-3, IL-1ß, VEGF, angiogenin, IL-8, ß2Defensin and ß3Defensin were detected in all subjects, Interferon-α was detected in none, while the remaining mediators were detected in 40% to 93% of subjects. Angiogenin, VEGF, FGF, IL-9, IL-7, lymphotoxin-α and IL-3 had not been previously reported in genital mucosal fluid from women. Strong correlations were observed between levels of TNF-α, IL-1ß and IL-6, between chemokines IP-10 and MIG and between myeloperoxidase, IL-8 and G-CSF. Samples from women with any STD/colonization had significantly higher levels of IL-8, IL-3, IL-7, IL-1ß, lactoferrin and myeloperoxidase. IL-1ß and lactoferrin were significantly increased in gonorrhea, *Chlamydia*, cervicitis, bacterial vaginosis and trichomoniasis.

**Conclusions/Significance:**

These studies show that mucosal fluid in general appears to be an environment that is rich in immune mediators. Importantly, IL-1ß and lactoferrin are biomarkers for STDs/colonizations providing insights into immune responses and pathogenesis at this mucosal site.

## Introduction

It is estimated that more than one million new cases of sexually transmitted diseases (STDs) occur each day worldwide [1]. STDs in women induce innate and/or adaptive immune responses that in most cases do not lead to clearance of the microbial infection, but can cause inflammation and/or influx of immune cells into the lower genital tract [2]. While not STDs, bacterial vaginosis and vaginal candidiasis are frequent microbial colonizations encountered in the setting of the STD clinic that also induce immune responses in the lower genital tract of women [3], [4]. The combination of the infecting/colonizing microorganisms along with the induced immune responses and inflammation can have serious pathologic consequences in women including infertility, adverse pregnancy outcomes, and increased susceptibility to infection by other pathogens [1], [5].

Identification of the immune mediators that are elicited in the lower genital tract by STDs and other microbial colonizations can potentially give important insights into the innate and adaptive immune pathways that are activated in response to the microorganisms, how the organisms are able to avoid clearance by these immune pathways as well as their pathologic mechanism. For example, some cytokines and chemokines are induced in the genital tract by STDs and this induction has been associated with increased susceptibility to infection with HIV suggesting a possible role for those immune mediators in increasing HIV susceptibility (Reviewed in [6]). Some genital immune mediators have been reported to be increased in trichomoniasis, *Chlamydia trachomatis* infection and in bacterial vaginosis [7], [8], [9]. However, most previous studies of STDs measured limited types of mediators (e.g. only proinflammatory mediators or chemokines) and the relationships between mediators and STDs have not been well defined.

In this study we tested genital mucosal fluid from women attending an STD clinic for 32 distinct immune mediators. The mediators measured in this study included pro-inflammatory cytokines (IL-1ß, TNF-α, IL-6), anti-inflammatory cytokines (IL-4, IL-10, IL-13), chemokines (MCP-1, RANTES, IL-8, IP-10, MIG, MIP-1α, MIP-1ß, eotaxin), anti-microbial proteins (ß2defensin, ß3defensin, lactoferrin), a marker for neutrophils (myeloperoxidase, MPO), interferons (interferon-α and interferon-γ), and other cytokines and growth factors (GM-CSF, G-CSF, IL-3, IL-12, IL-7, IL-5, vascular endothelial growth factor (VEGF), angiogenin, fibroblast growth factor (FGF), IL-9 and lymphotoxin-α). The goals of this study were to determine which of the immune mediators were detectable at this mucosal site, to determine if there were any strong associations between the different mediators, and to investigate the hypothesis that specific types or patterns of immune mediators would be broadly changed over all STDs or changed with particular pathogens. Such patterns could provide biomarkers predictive of pathogenesis and help identify immune responses important for immune control of pathogens.

## Methods

### Subjects

All studies were approved by the Institutional review boards of Rush University Medical Center and Cook County Stroger Hospital. The study population was recruited at the Ruth M. Rothstein CORE Center STD Screening Clinic of Cook County Stroger Hospital. Symptomatic, female patients presenting for an STD evaluation were approached for study participation and written informed consent was obtained from all subjects. Standard of care genital exams were performed and swabs taken for diagnosis. Cervical-vaginal lavage samples were then obtained by irrigation of the cervix with 10 mL of non-bacteriostatic sterile saline, followed by aspiration from the posterior fornix. Urine was also obtained for a pregnancy test. None of the subjects were pregnant. The following tests were performed; BD Probetec ET (Becton Dickinson, Franklin Lakes, NJ) for *Chlamydia trachomatis* and *Neisseria gonorrhoeae*; culture for herpes (serology was not run); for Syphilis a rapid plasma reagin test (Arlington Scientific, Utah) was performed and confirmed using passive particle agglutination (Fujirebio, Malvern, PA); for HIV, an enzyme immunoassay (Biorad Laboratories, Hercules, CA) was performed and confirmed by western blot; for Trichomonas, wet mount examination and ELISA for the p65 protein (HyTest, Turku, Finland) were both performed; for bacterial vaginosis, wet mount for clue cells, vaginal pH, whiff test, and Nugent gram stain were performed; for Candida a KOH preparation was examined microscopically; Pelvic Inflammatory Disease and Warts were diagnosed by clinical exam.

### Immunoassays

All immunoassays, except interferon-α, lactoferrin, myeloperoxidase (MPO), human ßdefensin2 (HßD2) and human ßdefensin3 (HßD3) utilized Cytometric Bead Arrays (BD Biosciences, San Jose, CA). The commercial arrays had lower limits of detection of 1–3 pg/ml. Custom cytometric bead arrays were made to detect interferon-α, lactoferrin and myeloperoxidase by coupling blank beads (BD CBA Functional Beads) with either rabbit antibody to interferon-α (US Biological, Swampscott, MA), rabbit antibody to lactoferrin (Biodesign International, Saco, ME) or rabbit antibody to myeloperoxidase (ICL Inc., Newberg, OR). Standards for the custom bead arrays were recombinant human interferon-α2 (Cell Sciences, Canton, MA), milk lactoferrin (Sigma Chem. Co, St. Louis, MO) and myeloperoxidase (Calbiochem EMD Chemicals, Gibbstown, NJ). Sensitivities for the interferon-a , lactoferrin and myeloperoxidase assays were 15 pg/ml, 30 pg/ml and 30 pg/ml respectively. All bead arrays were assayed on a FACS Calibur flow cytometer and levels of cytokines calculated using BD CBA software. HßD2 and HßD3 were measured by ELISA using previously described methods [10].

### Statistical Analysis

We first performed a summary analysis of the counts of STDs/conditions and the immune mediators on the 84 subjects. Correlation coefficients between pairs of the mediators were computed and the null hypothesis of no linear association was tested. Univariate logistic regression analyses were performed in examining the association between having STDs/conditions and the immune mediators or the clinical factors. Multivariate logistic regression with stepwise variable selection was then performed on the significant variables found in the univariate analysis. Subjects with a particular STD or condition were compared with those without any STD/condition by logistic regression when the number of cases was relatively large and by the Fisher exact test when the number of cases was small. Such analyses were performed both on subjects having the STD/condition alone and for all subjects having the STD/condition. No multivariate analysis was performed for individual STD/condition because of the sample size limitation.

## Results

### Subject characteristics

The study population consisted of 84 women, 18 years of age or older, attending a clinic for STD screening. The largest proportion of subjects were between 18 and 24 years old (40%), while 33% were between 25 and 34, 18% were between 35 and 44 and 8% were between 45 and 54. The subjects were 90% African American, 7% Latino, and 3% Caucasian.

Of the 84 subjects, 13 had no STD or condition (conditions included bacterial vaginosis and Candida that are technically not STDs), 49 subjects had one STD or condition, 20 subjects had 2 STDs or conditions while 3 subjects had 3 STDs or conditions. The numbers and types of STDs and conditions are shown in Table 1. The most common diagnosis was bacterial vaginosis followed by Candida, Chlamydia infection and trichomoniasis.

**Table 1:** Frequency of STDs and/or Conditions.

	Only	All
Bacterial Vaginosis	12	23
Yeast/Candida	12	22
Trichomoniasis	6	12
Chlamydia Trachomatis	5	13
Cervicitis	3	8
Gonorrhea Cervicitis	3	8
Lesions	2	2
Vaginitis	1	2
HSV	0	4
Warts	0	3
PID*	0	2
No Condition	13	13

*Pelvic Inflammatory Disease.

In the 48 hours prior to cervical-vaginal lavage (CVL) donation, one woman reported use of a vaginal tampon, five reported douching, nine reported use of vaginal medications (either suppositories, creams, jellies, foam, sponge, perfume or lubricant), 23 reported having vaginal sex with a male partner while 2 reported menstrual blood flow (although blood was not evident at the time of CVL donation). All CVL was tested for semen using the Abacard test for prostate specific antigen (PSA). Fourteen were PSA positive, three were PSA +/− while 67 were PSA negative.

### Detection of immune mediators

Table 2 shows the median, mean and range for each of the 32 immune mediators listed from lowest to highest median concentration in CVL samples. Six mediators were detected in all CVL (IL-1ß, VEGF, angiogenin, IL-8, HßD2 and HßD3) while interferon-α was the only substance not detected in any of the CVL samples. Lactoferrin, MPO and GCSF were the three most frequently detected of the remaining mediators (detected in 88%, 90% and 93% of subjects respectively) while lymphotoxin-α, IL-9 and eotaxin were the three least frequently detected (40% 45% and 46% respectively). Lymphotoxin-α, IL-13, IL-9, eotaxin, RANTES, IL-2, IFN-γ, IL-5 and MIP-1α were detected too infrequently to obtain reliable associations with other mediators or with disease status and therefore were excluded from the further analysis described below.

**Table 2:** Levels of Immune Mediators in CVL.

Mediator*	Missing	ND	Min	Median	Max	Detectable	Mean	SD
IFN-α	0	84	ND	ND	ND	0		
LT-α	0	51	ND	ND	14	33	3	2
IL-13	0	50	ND	ND	50	34	10	8.1
IL-9	0	47	ND	ND	74	37	15	12
IFN-γ	8	38	ND	ND	92	38	25	21
Eotaxin	3	46	ND	ND	573	35	84	100
IL-4	8	26	ND	1.8	20	50	4	4
IL-10	8	24	ND	2	23	52	5	5
RANTES	5	39	ND	3	178	40	17	30
IL-2	8	24	ND	3	20	52	5	4
TNF-α	8	24	ND	3	190	52	13	28
MIP-1 α	0	38	ND	3	162	46	17	32
IL-5	9	2	ND	3	14	73	4	2
IL-3	3	36	ND	3	89	45	10	13
G-CSF	3	6	ND	4	52	75	438	879
GM-CSF	3	17	4	52	64	7	7	
IL-6	8	13	ND	5	1736	63	93	312
IL-12	7	8	ND	8	81	69	12	12
MCP-1	5	20	ND	12	2500	59	80	327
MIP-1ß	0	16	ND	21	5714	68	183	729
FGF	0	41	ND	28	86	43	41	12
IL-7	9	18	ND	44	285	57	90	68
IP-10	5	17	ND	54	2500	62	328	590
MIG	5	5	ND	64	2500	74	391	661
IL-1ß	7	0	2.2	184	5082	77	610	1050
VEGF	0	0	33	246	15742	84	627	1813
Angiogenin	0	0	18	459	151800	84	3258	16644
IL-8	7	0	31	3729	5046	77	2909	2085
HBD2	0	0	1.6	7	35	84	9	6
MPO	0	8	ND	369	6400	76	904	1145
HBD3	0	0	1.1	1069	3156	84	1127	839
Lactoferrin	0	10	ND	1741	5198	74	1669	917

Table is arranged from lowest to highest median values. ND: Not Detectable; Mean and SD (Standard Deviation) are computed for the detectable.

The total sample size: N_0_+N_1_+N_2_ = 84.

*Levels in pg/ml except that HBD2, MPO, HBD3 and Lactoferrin are in ng/ml.

### Associations between mediators

A number of significant positive associations between the immune mediators were observed. Associations with Pearson correlation coefficients >0.7 (strong correlations) are shown in Table 3. The strongest correlation was between MIG and IP-10, two IFN-γ-induced, CXCR3-binding chemokines that also have direct anti-bacterial activity [11]. Strong correlations were also observed between each of the three pro-inflammatory cytokines TNF-α, IL-1ß and IL-6. MPO, a marker for the presence of neutrophils, was strongly correlated with IL-8 and G-CSF, mediators that induce neutrophil migration and support neutrophil function, respectively.

**Table 3:** Associations Between Mediators with Pearson Correlation Coefficients >0.7.

	TNF-α	IL-3	IL-6	IL-7	IL-8	IL-10	IL -1β	GM-CSF	IP-10	MIG	G-CSF	MPO
TNF-α	1.00		0.70			0.81	0.70					
IL-3		1.00						0.81				
IL-6			1.00			0.73	0.74				0.83	
IL-7				1.00								
IL-8					1.00		0.83				0.71	0.84
IL-10						1.00		0.77				
IL -1β							1.00				0.77	
GM-CSF								1.00				
IP-10									1.00			
MIG										1.00		
G-CSF											1.00	

The statistic is the correlation coefficient. Correlation coefficients smaller than 0.7 are suppressed. All displayed correlation coefficients have p-values smaller than 0.0001 in testing the hypothesis of no correlation.

No significant strong negative correlations (Pearson correlation coefficients between −1.0 and −0.7) were observed between any of the immune mediators possibly reflecting the fact that only a few of the mediators tested had known activity for down-regulating immune responses. However, significant (p<0.05), but relatively weak, negative correlations between HßD2 were observed with IL-8, IL-1ß, GM-CSF, IP-10, MIG, G-CSF and MPO (Pearson coefficients ranging from −0.24 to −0.34). Similarly, HßD3 had negative correlations with TNF-α, IL-4, IL-6, IL-10, IL-1ß, IL-12, GM-CSF, MCP-1, G-CSF, VEGF and lactoferrin (Pearson coefficients ranging from −0.24 to −0.42).

### Association of mediators with STD/colonization

The subjects that had no STDs or colonization were used as a control group and compared with all other subjects to determine if having any STDs/colonization was associated with changes in levels of immune mediators. When compared to controls, women with any STD/colonization had significantly higher levels of IL-3, IL-4, IL-6, IL-8, IL-10, IL-1ß, MIG, G-CSF, VEGF, Angiogenin, lactoferrin and MPO (Table 4). In a multivariate analysis however, only MIG and lactoferrin were significantly different between the two groups (Table 4).

**Table 4:** Relationships between STDs/Conditions and Immune Mediators.

Mediator*	Control (SD)	Any STD (SD)	Univariate Analysis			Multivariate Analysis		
			OR	95% CI	P	OR	95% CI	P
TNF-α (8)	3 (1)	10 (26)						
IL-4 (9)	2 (1)	3 (4)	2.0	1.1, 3.7	0.02			
IL-6 (7)	5 (5)	92 (308)	1.9	1.1, 3.3	0.02			
IL-10 (9)	2 (2)	4 (5)	2.1	1.1, 4.4	0.03			
IP-10 (5)	75 (27)	294 (573)						
MCP-1 (5)	24 (5)	67 (305)						
MIG (5)	87 (23)	421 (682)	1.5	1.1, 2.1	0.02	1.5	1.0, 2.1	0.04
IL-8 (7)	679 (219)	679 (219)	1.7	1.1, 2.6	0.01			
GM-CSF (3)	3 (2)	7 (8)						
G-CSF (3)	35 (12)	476 (902)	1.5	1.1, 2.1	0.008			
IL-3 (9)	12 (10)	24 (21)	7.4	1.5, 38	0.02			
IL-7 (9)	31 (22)	76 (74)						
IL-12 (7)	7 (7)	12 (13)						
IL-1ß (7)	72 (12)	719 (1103)	1.8	1.2, 2.6	0.003			
VEGF (0)	185 (164)	708 (1937)	2.4	1.1, 5.4	0.03			
Angiog. (0)	605 (201)	3744 (17829)	1.8	1.1, 3.1	0.03			
FGF (0)	27 (35)	20 (22)						
MIP-1ß (0)	29 (18)	170 (706)						
HBD2 (0)	10 (18)	9 (14)						
MPO (0)	217 (92)	928 (1183)	1.3	1.1, 1.7	0.02			
Lactof. (0)	370 (176)	1672 (959)	1.4	1.1, 1.7	0.002	1.3	1.1, 1.6	0.008
HBD3 (0)	1446 (1822)	1069 (1585)						

Levels in pg/ml except that HBD2, MPO, HBD3 and Lactoferrin are in ng/ml. Any STD includes BV and Candida. Only variables with p-value<0.05 have OR, 95% confidence interval and p-value shown. The p-values are calculated based on Fisher exact test and the odds ratios are calculated with correction, i.e., by adding 0.5 to counts if there were zero counts. Only two variables are selected in the final model.

*Number missing in parenthesis.

Subjects were further sub-categorized as having either no STD or colonization, only one STD/colonization or several STD/colonizations (Table 5). Compared to the control group, women with only GC, Chlamydia, cervicitis, bacterial vaginosis or Trichomoniasis had significantly different levels of 7, 17, 4, 11 and 9 different immune mediators respectively (Table 5). There were too few women with HSV, non-specific lesions, warts or PID to obtain meaningful comparisons with the control group. In general, when more than one STD/colonization was present (“All”, Table 5), more mediators were significantly different than controls (13, 17, 8, 15 and 5 for GC, Chlamydia, cervicitis, bacterial vaginosis (BV) and trichomoniasis repectively). Most of the significant differences were increases in mediators when compared to control. However, MIP-1ß was decreased in trichomoniasis, while HßD2 was decreased in Chlamydia infection. Strikingly, IL-1ß and lactoferrin were both significantly increased in all five of the STDs/colonizations. A varying pattern of significant changes in other mediators was seen. For example MIP-1α, VEGF and MPO levels were significantly different than controls in *Chlamydia* and trichomoniasis but not BV while in contrast, IL-6, IL-10, GM-CSF, G-CSF, IL-3, IL-7 and IL-12 were significantly changed in *Chlamydia* and BV but not trichomoniasis.

**Table 5:** P-values in testing association between specific STDs/Conditions and Immune Mediators.

	Gonorrhea		Chlamydia		Cervicitis		B. Vaginosis		Trichomonas	
	Only*	All*	Only	All	Only	All	Only	All	Only	All
TNF-α		0.02	0.03	0.008				0.006		
IL-4		0.02		0.01			0.02	0.01	0.04	
IL-6		0.007	0.004	0.001	0.01	0.03	0.03	0.004		
IL-10	0.02	0.01	0.001	0.001			0.01	0.01		
IP-10				0.01		0.04				0.03
MIG			0.04	0.003		0.03		0.03	0.01	0.01
IL-8	0.02	0.01	0.001			0.01	0.03	0.002	0.04	
GM-CSF		0.02	0.02	0.02			0.007	0.006		
G-CSF	0.01	0.001	0.0001	0.0001			0.02	0.009		
IL-3			0.05	0.04			0.003	0.004		
IL-7	0.01	0.002	0.02	0.006			0.02	0.002		
IL-12		0.02	0.02	0.004			0.001	0.0001		
IL-1ß	0.007	0.002	0.002	0.0001	0.02	0.005	0.001	0.0001	0.01	0.02
VEGF	0.03	0.02	0.002	0.003		0.004		0.001	0.03	
Angiog.		0.03	0.01	0.01				0.004		
MIP-1ß			0.02		0.04				0.006	0.05
Lacto.	0.002	0.0003	0.004	0.0001	0.003		0.001	0.001	0.001	0.03
MPO			0.02	0.003		0.002			0.04	
HBD2			0.02	0.003		0.002			0.04	

Only those p-values<0.05 are shown. Tests are significant after the adjustment of column-wise multiple tests if p-value<0.05/32 = 0.0016, where 32 is the number of tests performed, including those mediators tested but not shown.

*Only -subjects that had only one STD/colonization; All –all subjects with that STD/colonization.

MCP-1, FGF and HßD3 were not significantly changed in any of the STDs/colonizations, while MIG and IP-10 were not significantly different in any of the “only” STDs/colonizations.

**Figure 1:**
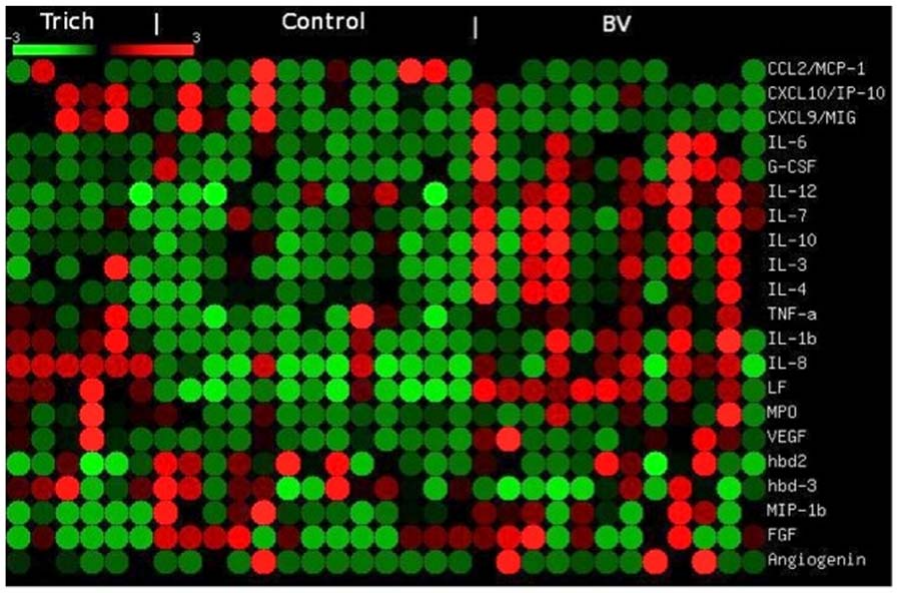
Heat map of cytokine levels. The heat map was generated using the web-based program Matrix2png [34] that displays microarray data visually (http://www.bioinformatics.ubc.ca/matrix2png/). The mean for each row of cytokine values is 0 with red representing values greater than 0, green lower than 0 and black 0.

When viewed in a heat map format, differential patterns of increases in cytokines in relation to disease were apparent when comparing levels from subjects with trichomoniasis, bacterial vaginosis and controls (Fig. 1). Thus, IL-1ß, lactoferrin and IL-8 were increased in both trichomoniasis and BV when compared to controls. In contrast, IL-12, IL-7, IL-10, IL-3, and IL-4 were increased in BV when compared to control samples but not in trichomoniasis.

Interestingly, women with only yeast infection (N = 12) did not have a significant change in any of the immune mediators (not shown).

## Discussion

This study provides several novel observations concerning immune mediators in genital mucosal fluids of women. First, IL-1ß and lactoferrin levels were significantly increased when comparing controls with all infected subjects and when comparing controls with each of the STDs/colonizations except Candida. Thus, increased levels of IL-1ß and lactoferrin were the most robust changes found in this clinic population with IL-1ß levels increased 10-fold and lactoferrin increased 4.5 fold compared to controls. IL-1ß is a strong inducer of inflammation at mucosal sites and IL1ß release from cells is mediated by the inflammasome, an intracellular molecular complex that is the subject of recent intense interest [12]. The inflammasome is activated not only by microbial products including peptidoglycans and toxins, but also by host-derived danger signals such as changes in potassium levels, extracellular ATP and reactive oxygen species suggesting that these endogenous and less-specific inducers could be the commonality that is present in all STDs/colonizations that leads to IL-1ß production [12]. Lactoferrin is an iron-binding protein synthesized by neutrophils and epithelial cells and is implicated in the host defense response against bacterial, fungal and viral pathogens [13], [14], [15]. Lactoferrin and IL-1ß are both associated with increased HIV-1 genital tract shedding in women [16]. Both lactoferrin and IL-1ß have been reported to be elevated in bacterial vaginosis and increased IL-1ß was found in chlamydia infection while increased lactoferrin was reported in cervicitis and trichomoniasis [7], [17], [18], [19], [20]. However, elevations of lactoferrin in Chlamydia infection and Gonorrhea have not been previously reported and elevated IL-1ß in trichomoniasis, Gonorrhea or cervicitis have not been previously reported. One study reported that IL-1ß was not elevated in *Trichomonas vaginalis*, *Chlamydia trachomatis* or *Neisseria gonorrhoeae* infections [21]. Lactoferrin also is recognized to be anti-inflammatory and in fact has been shown to down-regulate IL-1ß-induced inflammation in the skin [22]. Therefore, lactoferrin in the genital tract may counteract the pro-inflammatory environment induced by IL-1ß and other cytokines. Lactoferrin was previously found to be associated with leukocyte levels in CVL samples [23] and in the current study, lactoferrin was significantly associated with MPO levels (p<0.0001) although the correlation coefficient of 0.47 did not achieve the level of the strong correlations shown in Table 3. In microbicide studies, IL-1ß is considered a marker for inflammation/damage to the epithelium [24].

Other potentially important findings of this study were that mucosal fluid in general appears to be an environment that is rich in immune mediators, since all of the 32 mediators that were tested, except one, were detected in at least 40% of the subjects and several (IL-3, IL-1ß, VEGF, Angiogenin, IL-8, ß-Defensins 2 and 3), were detected in all of the subjects. To our knowledge, some of the mediators, including VEGF, angiogenin, FGF, IL-9, IL-7, lymphotoxin-α and IL-3, had not been previously reported to be present in female genital secretions. While the role that these previously-unrecognized mediators play in genital immunity is not known, several have reported functions that could be important in immune responses to STDs. For example, angiogenin has anti-microbial activity against *Candida albicans* and certain bacteria in vitro [25], but has not yet been tested for a possible role in immunity to genital yeast or other infections in women. VEGF has been postulated to play a role during inflammation and infections by increasing vascularity and promoting adhesion of leukocytes [26], [27]. IL-7 is a crucial survival and expansion factor for T cells [28] and could therefore play a role in mucosal T cell responses to STDs.

This study also showed strong correlations between several groups of immune mediators, suggesting possible linkages of inducing pathways or common producing cell types. IP-10 and MIG had the strongest association and these two chemokines have many previously described parallels. They are both induced by interferons, bind to CXCR3, have direct anti-bacterial activity and are antagonists for CCR3 [11], [29]. Strong correlations were also observed between each of the three pro-inflammatory cytokines TNF-α, IL-1ß and IL-6 (Table 2). IL-8, which is in some cases considered a pro-inflammatory mediator, was also strongly associated with IL-1ß, but not with TNF-α and IL-6. Several previous studies have noted parallels between several of these inflammatory mediators in genital fluid from different groups of women. For example, levels of IL-1ß and IL-6, but not TNF-α, were significantly elevated in subjects in labor when compared to those not in labor [30]. In contrast, several studies have shown that while IL-1ß is increased in bacterial vaginosis, IL-6 and TNF-α are not [7], [31]. In this study, IL-1ß, IL-6 and IL-8 were significantly increased in bacterial vaginosis while TNF-α was not.

This study had several limitations. Several conditions in the subjects were not known at the time of sample collection such as smoking and exact stage of the menstrual cycle which have been shown to be associated with changes in levels of certain cytokines [32]. Also, the women used as a control group were those visiting the STD clinic. Thus it is possible that a group of women with no risk factors may have had differing levels of immunologic mediators than the group assessed here. Another limitation of this study was that samples were collected by cervical vaginal lavage and therefore the initial concentration of mediators in undiluted genital fluid is not known. However, collection of samples by this method would not affect the associations between mediators that were observed. Further, the numbers of women infected with herpes are likely to be underestimated in this group of women since culture was used to determine herpes infection instead of the more sensitive PCR-based methods [33].

In conclusion this study shows that IL-1ß and lactoferrin are increased in a broad array of STDs and other genital conditions potentially providing insights into pathogenesis and immune responses at this mucosal site. These studies also show that genital mucosal fluids in women have a wider range of immune mediators than previously demonstrated.
